# Comparison of Spermatozoa Quality in Male Smokers and Nonsmokers of Iranian Infertile Couples

**Published:** 2011-12-22

**Authors:** Mahshid Aryanpur, Majid Tarahomi, Hooman Sharifi, Gholamreza Heydari, Zahra Hessami, Mohamadmehdi Akhoundi, Mohamad Reza Masjedi

**Affiliations:** 1 Tobacco Prevention and Control Research Center (TPCRC), National Research Institute of Tuberculosis and Lung Diseases, Shahid Beheshti University of Medical Sciences, Tehran, Iran; 2 Reproductive Biotechnology Research Center, Avicenna Research Institute, ACECR, Tehran, Iran; 3 National Research Institute of Tuberculosis and Lung Diseases, Shahid Beheshti University of Medical Sciences, Tehran, Iran

**Keywords:** Semen Analysis, Smoking, Infertility, Sperm Concentration

## Abstract

**Background:**

Smoking has a negative effect on fertility and sperm quality. This study was designed to evaluate the effects of smoking on sperm quality and the related parameters such as sperm concentration, morphology and motility.

**Materials and Methods:**

This cross-sectional study was conducted on 180 infertile men with at least one year history of idiopathic infertility, who admitted to the Avicenna Infertility Center, Tehran, Iran. A complete history including smoking habits and other diseases was obtained and semen analysis was performed for all participants. Statistical analysis was performed using SPSS software version 16 and t test and Mann-whitney tests with a significance level of α= 0.05.

**Results:**

Comparison of sperm parameters in the two groups of smoker and nonsmoker subjects showed that active smoking (p=0.04) and cigarette consumption even in small amounts (p=0.03) decreased sperm concentration, However, no significant correlation was detected between smoking status and morphology or motility of sperms.

**Conclusion:**

This study failed to find a significant correlation between sperm analysis and smoking status except for sperm concentration, which was significantly decreased in the active smokers ,even in those consuming small amounts of tobacco. This finding propounds that tobacco consumption may negatively affect fertility.

## Introduction

Despite the efforts made in order to control tobacco consumption across the world, smoking is still common (1). It is considered as a prevalent hazardous social habit and is more common among men compared to women (2). The highest prevalence of smoking is seen among young men during fertility years and 46% of world smokers are in the age range of 20-39 years (1). In Iran, 24.1% of men and 4.3% of women over the age of 15 are active smokers and this rate is increasing (3). Studies on infertile couples in Iran have shown that,despite the negative effect of smoking on fertility, 20.6% of infertile couples (37.7% of men and 3.5% of women) still smoke (4).

Cigarette smoke contains known mutagenic and carcinogenic substances, and is believed to have a significant negative effect on male fertility (2). Among the infertile couples, 35% of the infertility is due to man and 25% is due to both partners (5). In the case of male problem, the cause is found in only 40% of the cases and in the rest of 60% of the time, it remains pathologically unknown (6). Therefore, it is important to study the factors affecting male fertility. Smoking decreases sperm concentration and fertility in men. Adverse effect of smoking on semen quality has been supported by many studies; but some are still inconclusive (7).

Cigarette smoking has negative effects on male fertility. Recent studies have shown the active transfer of several components of cigarette smoke through the blood-testis barrier (8). The presence of these components in the seminal plasma may induce degradation of sperm parameters and nuclear quality of spermatozoa. They may also compromise the chances of pregnancy. Moreover, smoking may have a negative impact on the smokers offspring and may result in poor quality embryos or development of childhood cancers. Oxidative stress-induced DNA damage seems to be one of the major causes of sperm quality alteration (8).

Smoking is associated with a small reduction in semen quality including sperm concentration, motility and morphology. It is also associated with alterations in hormone levels in males. for example, it increases the levels of oestrone and oestradiol.Male smokers with estron quality who wish to have children may benefit from quitting smoking (9).

Some studies have reported the negative effect of smoking on sperm concentration (10- 13), motility (12- 15) and morphology (10). They have also supported the adverse effect of smoking on all parameters of sperm quality (16). A meta-analysis of 27 studies revealed an average decrease of about 13% in sperm concentration, 10% in motility and 3% change in morphology (17). However, other studies did not report any impact on the parameters of spermatozoa quality (18, 19).

For example, in an Australian prospective study, the semen parameters and hormone concentrations of infertile smokers were compared with those of infertile non- and ex-smokers (men who had stopped smoking more than six months prior to the study). They studied 517 non-smokers, 109 exsmokers and 478 smokers, and found that smoking does not affect the conventional semen parameters, result of the study showed significantly increase of round and leukocytes (20).

The result of a study of semen quality was conducted on 197 smoker and 161 non-smoker Mexican males, undergoing initial infertility investigation, compared with non-smokers, smokers had significantly poorer sperm density, a lower percentage of viability, a lower percentage of sperms with normal morphology, and a lower percentage of motile sperms. These parameters were worse in the heavy smoker groups (21).

In another study, the semen quality of two groups of Italian men with idiopathic infertility (118 smokers and 153 nonsmokers) was compared. The infertile smoker and non-smoker patients showed similar sperm parameters. Although morphology evaluated by the transmission electron microscopy (TEM) analysis in both groups were significantly impaired compared with the controls, however sperm concentration and fertility index in the heavy smokers were significantly lower than those observed in the mild smoker and non-smoker groups (22).

This study was designed to evaluate the effect of smoking on concentration, motility and morphology of sperms in Iranian infertile couples.

## Materials and Methods

In this cross-sectional study performed from November 2008 to March 2009 at the Avicenna Infertility Clinic(Tehran, Iran),180 married men with one year history of idiopathic infertility who were able to produce semen, were randomly selected to participate in the study . This survey was approved by the Ethics Committee of National Research Institute of Tuberculosis and Lung Diseases (NRITLD) and the patients satisfaction was documented.

Cases with a history of cryptorchidism, orchitis, varicocele, inguinal hernia, testicular trauma, vasectomy, systemic or urogenital diseases, exposure to a specific substance or drug consumption were excluded from the study.

Smoking history was evaluated through a standard questionnaire containing questions on smoking status, number of cigarettes smoked per day, years of smoking, nicotine dependence and wife’s smoking status.

Patients with a history of smoking were considered smokers and those with no history of smoking were considered nonsmokers. Based on the World Health Organization (WHO) standards, if one had smoked 100 cigarettes in his life time, he was considered an active smoker (if a participant smoked at least one cigarette per day, he was considered as daily smoker, if he smoked occasionally during last year, he was categorized as occasional smoker and if one had smoked 100 cigarettes in his life time, but quitted and was a non-smoker at the time of study, he was considered as an ex-smoker).

Nicotine dependence was evaluated using the Fagerstrom’s test, which contains 6 questions with a score of 0 -10, and based on their score, the smokers were grouped into mild (score,0-3), moderate (4- 6) or severe dependence (7- 10).

The semen samples were collected based on the WHO standards after 3-6 days of abstinence (23).

Microscopic examination and evaluations of the pH, volume, viscosity, appearance, concentration, motility, agglutination, and morphology analysis of sperms were all performed.

The results obtained were evaluated based on theWHO criteria as follow:

Semen volume -(ml): minimum of 1.5 ml
Sperm concentration – (sperm per ml): minimum of 15 million/ml
Total motility: minimum of 40%
Morphology (normal forms): minimum of 4%.

Descriptive analyses were performed using the SPSS software, version 16. T test and Mann - Withney tests with a significance level of α=0.05 were also used for statistical analysis. Kolmogorov Smirnov’s test, normal curves and histograms were used to determine distribution normality. If distribution was not normal, Mann - Withney test was employed. Using t test, the variables were placed into groups of smoker and nonsmoker and also active smoker, and the remaining group (the remaining group contained occasional smokers plus nonsmokers. Ethical issues like privacy of information were followed in this study and an informed consent was obtained from all the participants.

## Results

One hundred and eighty individuals presenting to the Avicenna Infertility Center participated in this study.The cases were in the age range of 22 to 68 years with a mean age of 35.26 ± 6.63 years. A total of 56 (31.3%) participants had high school diploma and 46 (25.6%) had collage education or Bachelor’s degree. A total of 69 subjects (38.3%) were employers, 47 (26.1%) were employees , 41(22.7%) were workers and 23(12.7%) had other occupations. Duration of treatment for infertility ranged from 1 month to 25 years with a mean of 4.86 ± 5.4 years.

Also 68 (37.8%) subjects were nonsmokers and 112 (62.2%) were smokers among them71 (39.4%) subjects were active smokers. Of these 71 cases, 21 (11.7%) had quitted smoking by the time of conduction of this study and the remaining 50 (27.8%) continued to smoke actively. Of the latter, 14 (7.8%) smoked occasionally and 36 (20%) smoked daily (Table 1).

Sperm morphology results showed that 168 individuals (93.3%) were teratozoospermic. Sperm motility evaluation revealed that 60 individuals (33.3%) were astenozoospermic (Table 2).

**Table 1:** Distribution of active smoking status (number of cigarettes per day early morning smoking and nicotine dependence) among the infertile married men presenting to the Avicenna Infertility Center


	Active smoking	Number	Percentage

**Number of cigarettes smoked daily**	Less than 10 cigarettes	31	64.6%
	10-30 cigarettes	6	12.5
	More than 30 cigarettes	11	22.9
	Total	48	100
**Early morning smoking**	In the first 5 minutes	4	8.3
	In 6-60 minutes after waking up	5	10.4
	One hour after waking up	39	81.3
	Total	48	100
**Nicotine dependence**	Mild dependence	31	64.6
	Moderate dependence	10	20.8
	Severe dependence	7	14.6
	Total	48	100


**Table 2:** Distribution of the sperm analysis parameters (minimum, maximum, mean) among the infertile married men presenting to the Avicenna Infertility Center


Semen analysis	Minimum	Maximum	Mean

**Concentration**	10	580×10^6^	84 × 10^6^ ± 78 × 10^6^
**Normal morphology**	0%	42%	11.35% ± 8.22%
**Motility**	0%	80%	49.97% ± 17.49%


**Table 3:** Distribution of the mean sperm characteristic based on the smoking status among the infertile married men presenting to the Avicenna Infertility Center


Semen analysis	Sperm concentration	Morphology	Motility

**Non-smoker**	102×10^6^ ± 100×10^6^	10.39 ± 8.09	49.55 ± 15.17
**Smoker**	73×10^6^ ± 58×10^6^	11.94 ± 8.27	50.22 ± 18.82
**P value**	0.03	0.21	0.4
**The remaining group**	94×10^6^ ± 90×10^6^	10.76 ± 8.24	49.85 ± 16.64
**Active smoker**	68×10^6^ ± 51×10^6^	12.28 ± 8.16	50.15 ± 18.83
**P value**	0.04	0.24	0.64


Sperm analysis abnormalities were more common in the smokers and active smokers compared to the non-smokers. T test and chi square put: Mann-whitney tests were used to compare sperm concentration, morphology and motility based on the smoking status. The mean sperm concentration was 102×10^6^ ± 100×10^6^ in the nonsmokers and 73, ×10^6^ ± 58×10^6^ in the smokers, which was significantly lower in smokers (p=0.03). No significant difference was found between the smoking status and the sperm morphology or motility (Table 3).

T test and Mann - Whitney tests were done among the active smokers and the remaining group (nonsmokers plus occasional smokers). The mean sperm concentration of semen was 94×10^6^ ± 90×10^6^ and 68×10^6^ ± 51×10^6^ in the above mentioned groups, respectively and was statistically lower among the active smokers (p=0.04). However, no significant difference was observed in other semen characteristics (morphology and motility) between the two groups (Table 3).

## Discussion

Despite the harmful effects of smoking on humans health and being among the most important causes of morbidity and mortality worldwide, still 1/3 of the world population above 15 years of age smoke daily (3). In this study, 27.8% of the men continued to actively smoke compared to 24.1% rate reported by the WHO (3) and 27.2% rate reporterd in Iranian males (24). However, this rate was not significantly different from that of Iranian fertile men. In a study conducted in the United State on 87 infertile men, smoking prevalence was reported to be 38% (25) which is much higher than the rate we found in our study. This difference may be related to the effect of cultural and social factors between the two nations or decreased self-report of this habit by our under study population.

In this study, 20% of the infertile men smoked daily, 7.8% smoked occasionally and 11.7% were ex-smokers in comparison to the public statistics of 22% daily smokers, 2.5% occasional smokers and 12.3% ex-smokers. In contrast to the assumption that infertile men smoke less, statistics showed that their smoking rate was not significantly different from the general public. Further studies are required to determine whether smoking is a risk factor for infertility and decreased response to treatment.

Many studies have proposed a correlation between smoking and changes in semen quality but various results have been obtained. Important variables affected sperm concentration, morphology and motility (26- 29).

In some studies, decreased sperm concentration has reported to be in relation with smoking (10, 12, 15, 30). Based on a study leads to a significant change in sperm concentration (31). Also in a study by Kunzell and colleagues, decreased semen concentration was shown among the smokers (32). In a study by Mehrania in Iran, decreased sperm concentration was observed in the heavy smokers compared to the nonsmokers (33). Yet, there are some contradictory reports as well (7, 10, 12, 15). Osser and colleagues did not find a significant correlation between smoking and sperm concentration changes (30) and in studies by Hessa et al.(34), Trummer et al. (17) and Ozgur et al. (7),found no correlation was found between smoking and changes in sperm concentration.

In our study on Iranian male population, sperm concentration of the smokers was significantly lower than nonsmokers. This finding is in accordance with those of some other studies propounding the probability of racial or genetical susceptibility to nicotine: the fact is that in some nationalities, nicotine can influence sperm parameters more effectively. Also in the present study, a significant sperm concentration difference was found between the active smokers and the remaining group indicating the negative effect of even small amounts of tobacco consumption. Further studies are recommended on this subject.

Similarly, various results have been found regarding the changes in sperm morphology based on smoking status (7, 31, 32). Our study did not support sperm morphology and motility changes based on smoking status like other studies (17, 30- 35).

In the present study, most of the active smokers had mild nicotine dependence and smoked less than 10 cigarettes per day. However, there was a small correlation between the two factors, which suggesting that infertile smokers are trying to cut down smoking, or under-report their consumption.

As mentioned earlier, most of our study population were university graduates. This rate is higher than the educational level of Iranian general population. Therefore according this research, it seems that some factors related to educational level can impact the increased risk of male infertility, which should be further evaluated in an especially designed survey.

Smoking even in small amounts like one-time smoking per day may decrease the sperm concentration. Therefore, smoking cessation programs are recommended at least for the infertile patients. Other study with long-term fallow-up may clear the contradictory results observed in the literature.

## Conclusion

This study did not support an overall correlation between sperm analysis and smoking status. But sperm concentration was significantly decreased in active smokers and even in those consuming small amounts of tobacco product. So it suggest that tobacco consumption may negatively affect fertility.
